# Supplementary material for: Heralded Quantum Entanglement between Distant Matter Qubits
Source: Sci Rep. 2015 Jun 4;5:10110. doi: 10.1038/srep10110 (PMC4455230; doi:10.1038/srep10110)
Supplement: Supplementary Information [file srep10110-s1.pdf]

Supplementary Material: Heralded Quantum Entanglement between Distant Matter Qubits

Wen-Juan Yang[1,2], Xiang-Bin Wang[1,2,3*]
[1] State Key Laboratory of Low Dimensional Quantum Physics, Tsinghua University, Beijing 100084, People's Republic of China
[2] Synergetic Innovation Center of Quantum Information and Quantum Physics, University of Science and Technology of China, Hefei, Anhui 230026, China
[3] Shandong Academy of Information and Communication Technology, Jinan 250101, People's Republic of China

(Dated: March 2, 2015)

I. COMPLETE DERIVATION OF EQ. (14)

If we take the atom bath interaction into consideration initially, then the Hamiltonian of system and bath is

$$\begin{aligned}
H_A &= H_{S_A} + H_R + H_{SR_A}, \\
H_{S_A} &= \omega_e |e\rangle_{AA}\langle e| + \sum_{i=L,R} \omega_c a^\dagger_{i_A} a_{i_A} + \sum_{i=L,R} (g_1 a_{i_A} |e\rangle_{AA}\langle g_i| + H.c.), \\
H_R &= \sum_{i=L,R} \int_{-\infty}^{\infty} \omega b_i^\dagger(\omega) b_i(\omega) d\omega + \sum_{i=L,R} \int_{-\infty}^{\infty} \omega d_i^\dagger(\omega) d_i(\omega) d\omega, \\
H_{SR_A} &= \sum_{i=L,R} \int_{-\infty}^{\infty} \sqrt{\frac{\kappa_1}{2\pi}} (-i b_i(\omega) a^\dagger_{i_A} + H.c.) d\omega + \sum_{i=L,R} \int_{-\infty}^{\infty} \sqrt{\frac{\frac{\gamma_1}{2}}{2\pi}} (-i d_i(\omega) |e\rangle_{AA}\langle g_i| + H.c.) d\omega.
\end{aligned} \tag{S1}$$

The master equation is

$$\dot{\hat{\rho}}_{S_A}(t) = (\mathcal{C} + \mathcal{D}) \hat{\rho}_{S_A}(t), \tag{S2}$$

where superoperators $\mathcal{C}$ and $\mathcal{D}$ become

$$\begin{aligned}
\mathcal{C}\Omega &= -i[H_{S_A}, \Omega] - \sum_{i=L,R} \frac{\kappa_1}{2} \{a^\dagger_{i_A} a_{i_A}, \Omega\} - \frac{\gamma_1}{2} \{|e\rangle_{AA}\langle e|, \Omega\}, \\
\mathcal{D}\Omega &= \sum_{i=L,R} \kappa_1 (a_{i_A} \Omega a^\dagger_{i_A}) + \sum_{i=L,R} \frac{\gamma_1}{2} (|g_i\rangle_{AA}\langle e| \Omega |e\rangle_{AA}\langle g_i|),
\end{aligned}$$

given any operator Ω. Substituting Eq. (4) into Eq. (S2) we can get two separate equations

$$\dot{\hat{\rho}}_0 = \mathcal{D}\hat{\rho}_1 \tag{S3}$$

$$\dot{\hat{\rho}}_1 = \mathcal{C}\hat{\rho}_1 \tag{S4}$$

According to the definition of superoperators, we know that

$$\mathcal{C}\rho_1 = -i[H_{S_A}, \rho_1] - \frac{\kappa_1}{2} \sum_{i=L,R} \{a^\dagger_{i_A} a_{i_A}, \rho_1\} - \frac{\gamma_1}{2} \{|e\rangle_{AA}\langle e|, \rho_1\} \tag{S5}$$

and $\mathcal{D}\rho_1 = \sum_{i=L,R} \kappa_1 (a_{i_A} \rho_1 a^\dagger_{i_A}) + \sum_{i=L,R} \frac{\gamma_1}{2} (|g_i\rangle_{AA}\langle e| \rho_1 |e\rangle_{AA}\langle g_i|)$. It is easy to see that Eq.(S5) is equivalent to

$$i\frac{d}{dt} |\tilde{\psi}(t)\rangle_{in} = H_{eff_A} |\tilde{\psi}(t)\rangle_{in}, \tag{S6}$$

where the non-Hermitian effective Hamiltonian H_{eff_A} is

$$H_{eff_A} = H_{S_A} - i \sum_{i=L,R} \frac{\kappa_1}{2} a^\dagger_{i_A} a_{i_A} - i \frac{\gamma_1}{2} |e\rangle_{AA}\langle e|. \tag{S7}$$

II. DETAILED DERIVATION OF EQ. (24)

Now consider the case that the photon is initially outside the cavity B. We divide the whole process of photon injection into many infinitely short terms. At any time, the state is a linear superposition of an intra-cavity state $|\tilde{\psi}\rangle_{in}$ where the space outside the cavity is vacuum and a non intra-cavity state $|\varphi\rangle$ where there is one photon outside the cavity. For the intra-cavity state, we directly use the time evolution operator $\exp(-iH_{eff_B}t)$ (see Lemma 1). The main idea is this: Consider the input photon as a wave train. Part of the wave train will enter cavity B later in the time evolution. The part inside cavity B will evolve by Lemma 1. At different times there will be different parts of the wave train entering the cavity and each of them evolve according to Lemma 1 since the time points they entered the cavity. The whole intra-cavity state will be the summation of all these, i.e., integration of all these. Consider the following Hamiltonian of system B and its reservoir

$$H_B \;=\; H_{S_B} + H_R + H_{SR_B}, \tag{S8}$$

where

$$\begin{aligned}
H_{S_B} &= H_{0_B} + H_{int_B},\\
H_{0_B} &= \omega_e|e\rangle_{BB}\langle e| + \sum_{i=L,R}\omega_c a_{i_B}^\dagger a_{i_B},\\
H_{int_B} &= \sum_{i=L,R}(g_2 a_{i_B}|e\rangle_{BB}\langle g_i| + H.c.),\\
H_R &= \sum_{i=L,R}\int_{-\infty}^{\infty}\omega b_i^\dagger(\omega)b_i(\omega)d\omega,\\
H_{SR_B} &= \sum_{i=L,R}\int_{-\infty}^{\infty}\sqrt{\frac{\kappa_2}{2\pi}}(-ib_i(\omega)a_{i_B}^\dagger + H.c.)d\omega.
\end{aligned}$$

The initial state is $b_i^\dagger(\omega)|g_L\rangle_B|0\rangle$ where $|0\rangle$ denotes the vaccum state for both inside and outside cavity. In the rotating frame of $H_{0_B}+H_R$, the Hamiltonian of the whole system is:

$$V(t) \;=\; \sum_{i=L,R}[(g_2 e^{-i(\omega_c-\omega_e)t}a_{i_B}|e\rangle_{BB}\langle g_i| + H.c.) + \int_{-\infty}^{\infty}\sqrt{\frac{\kappa_2}{2\pi}}(-ie^{-i(\omega-\omega_c)t}b_i(\omega)a_{i_B}^\dagger + H.c.)d\omega]. \tag{S9}$$

The time evolution operator with $\triangle t$ is $Te^{-i\int V(t)dt}$ where T is the time order operator. For infinitely small Δt we make the $Te^{-i\int V(t)dt} = e^{-i\int_0^t V(t)dt}$. The system state is:

$$\begin{aligned}
|\psi'(\triangle t)\rangle &= \sum_{i=L,R}Te^{-i\int_0^{\triangle t}V(t)dt}b_i^\dagger(\omega)|g_L\rangle_B|0\rangle = \sum_{i=L,R}Te^{-i\int_0^{\triangle t}V(t)dt}b_i^\dagger(\omega)Te^{i\int_0^{\triangle t}V(t)dt}|g_L\rangle_B|0\rangle\\
&= \sum_{i=L,R}[b_i^\dagger(\omega) - \triangle t\sqrt{\frac{\kappa_2}{2\pi}}e^{-i(\omega-\omega_c)\triangle t}a_{i_B}^\dagger]|g_L\rangle_B|0\rangle.
\end{aligned} \tag{S10}$$

Transforming back to the Schrödinger picture, state should be

$$|\psi'(\triangle t)\rangle \;=\; \sum_{i=L,R}[e^{-i\omega\triangle t}b_i^\dagger(\omega) - \triangle t\sqrt{\frac{\kappa_2}{2\pi}}e^{-i\omega\triangle t}a_{i_B}^\dagger]|g_L\rangle_B|0\rangle. \tag{S11}$$

The second term on the right hand side of the equation represents the intra-cavity mode with a vacuum bath. As shown earlier in subsection "Entanglement between Atom A and Photon", the time evolution operator for this part should be simply $-e^{-iH_{eff_B}t}\triangle t\sqrt{\frac{\kappa_2}{2\pi}}e^{-i(\omega-\omega_c)\triangle t}a_{i_B}^\dagger|g_L\rangle_B|0\rangle$. At the time point of $2\triangle t$, the state becomes,

$$|\psi'(2\triangle t)\rangle \;=\; \sum_{i=L,R}\{e^{-2i\omega\triangle t}b_i^\dagger(\omega)|g_L\rangle_B|0\rangle - \triangle t\sqrt{\frac{\kappa_2}{2\pi}}[e^{-2i\omega\triangle t} + e^{-i\omega\triangle t}e^{-iH_{eff_B}\triangle t}]a_{i_B}^\dagger|g_L\rangle_B|0\rangle\}. \tag{S12}$$

For time $n\triangle t$ the state should be

$$|\psi'(n\triangle t)\rangle \;=\; \sum_{i=L,R}(e^{-in\omega\triangle t}b_i^\dagger(\omega)|g_L\rangle_B|0\rangle - \triangle t\sqrt{\frac{\kappa_2}{2\pi}}\sum_{j=0}^{n-1}e^{-i\omega(n-j)\triangle t}e^{-iH_{eff_B}j\triangle t}a_{i_B}^\dagger|g_L\rangle_B|0\rangle). \tag{S13}$$

Change sum to integral and neglect small quantity then at time t the state is

$$|\psi'(t)\rangle = \sum_{i=L,R}(e^{-i\omega t}b_i^\dagger(\omega)|g_L\rangle_B|0\rangle - e^{-iH_{eff_B}t}\int_0^t\sqrt{\frac{\kappa_2}{2\pi}}e^{-i\omega t'}e^{iH_{eff_B}t'}a_{i_B}^\dagger|g_L\rangle_B|0\rangle dt'). \quad \text{(S14)}$$

III. EXPLICIT FORMULAS OF EQ. (24)

The explicit formulas of Eq. (24) in the main text are:

$$\begin{aligned}
C_{1,\omega}(t) &= -\sqrt{\kappa_2}(\frac{\alpha(-\eta^2+\lambda^2+4\lambda\rho-4\rho^2)}{4(\eta-\lambda)(\eta+\lambda)\rho}+\frac{\alpha e^{2\rho t}}{4\rho}+2\alpha e^{\lambda t}\cdot\frac{2\eta(-\lambda+\rho)\cosh\eta t+(\eta^2+\lambda(\lambda-2\rho))\sinh\eta t}{4\eta(\eta-\lambda)(\eta+\lambda)}),\\
C_{2,\omega}(t) &= -\sqrt{\kappa_2}\frac{\beta}{2\rho}(e^{2\rho t}-1),\\
C_{3,\omega}(t) &= 0,\\
C_{4,\omega}(t) &= -\sqrt{\kappa_2}(\frac{\alpha(\eta^2-\lambda^2+4\lambda\rho-4\rho^2)}{4(\eta-\lambda)(\eta+\lambda)\rho}-\frac{\alpha e^{2\rho t}}{4\rho}+2\alpha e^{\lambda t}\cdot\frac{2\eta(-\lambda+\rho)\cosh\eta t+(\eta^2+\lambda(\lambda-2\rho))\sinh\eta t}{4\eta(\eta-\lambda)(\eta+\lambda)})
\end{aligned} \quad \text{(S15)}$$

IV. EXPLICIT FORMULAS OF EQ. (28)

The explicit formulas of Eq. (28) in the main text are:

$$\begin{aligned}
cc_1(t) &= \sum_{i=1,2}(-1)^{i+1}s\{e^{-\Gamma_i t}\frac{\kappa_2 g_2^2+\kappa_2(\Gamma_i-\frac{\kappa_2}{2})(\Gamma_i-\frac{\gamma_2}{2})}{[|\eta|^2+(\frac{\kappa_2+\gamma_2}{4}-\Gamma_i)^2](\frac{\kappa_2}{2}-\Gamma_i)}+e^{-\frac{\kappa_2+\gamma_2}{4}t}\kappa_2 g_2^2[\frac{[|\eta|^2-(\frac{\kappa_2+\gamma_2}{4}-\Gamma_i)(\frac{\gamma_2-\kappa_2}{4})]\sin(|\eta|t)}{|\eta|[|\eta|^2+(\frac{\kappa_2+\gamma_2}{4}-\Gamma_i)^2][|\eta|^2+(\frac{\gamma_2-\kappa_2}{4})^2]}\\
&\quad-\frac{(\frac{\gamma_2}{2}-\Gamma_i)\cos(|\eta|t)}{[|\eta|^2+(\frac{\kappa_2+\gamma_2}{2}-\Gamma_i)^2][|\eta|^2+(\frac{\gamma_2-\kappa_2}{4})^2]}]-e^{-\kappa_2 t}\frac{\kappa_2 g_2^2}{[|\eta|^2+(\frac{\gamma_2-\kappa_2}{4})^2](\frac{\kappa_2}{2}-\Gamma_i)}-e^{-\Gamma_i t}\},\\
cc_2(t) &= \sum_{i=1,2}(-1)^{i+1}s[-\frac{\kappa_2(e^{-\frac{\kappa_2}{2}t}-e^{-\Gamma_i t})}{\frac{\kappa_2}{2}-\Gamma_i}-e^{-\Gamma_i t}],\\
cc_3(t) &= \sum_{i=1,2}(-1)^{i+1}s\{-e^{-\Gamma_i t}\frac{\kappa_2 g_2^2}{[|\eta|^2+(\frac{\kappa_2+\gamma_2}{4}-\Gamma_i)^2](\frac{\kappa_2}{2}-\Gamma_i)}+e^{-\frac{\kappa_2+\gamma_2}{4}t}\kappa_2 g_2^2[\frac{[|\eta|^2-(\frac{\kappa_2+\gamma_2}{4}-\Gamma_i)(\frac{\gamma_2-\kappa_2}{4})]\sin(|\eta|t)}{|\eta|[|\eta|^2+(\frac{\kappa_2+\gamma_2}{4}-\Gamma_i)^2][|\eta|^2+(\frac{\gamma_2-\kappa_2}{4})^2]}\\
&\quad-\frac{(\frac{\gamma_2}{2}-\Gamma_i)\cos(|\eta|t)}{[|\eta|^2+(\frac{\kappa_2+\gamma_2}{2}-\Gamma_i)^2][|\eta|^2+(\frac{\gamma_2-\kappa_2}{4})^2]}]+e^{-\kappa_2 t}\frac{\kappa_2 g_2^2}{[|\eta|^2+(\frac{\gamma_2-\kappa_2}{4})^2](\frac{\kappa_2}{2}-\Gamma_i)}\}.
\end{aligned} \quad \text{(S16)}$$

Here we denote $s=\frac{\sqrt{2}i\nu(\nu^2-\mu^2)}{\sqrt{\kappa_1}\mu g_1}$, $\Gamma_1=-(\nu+\mu)$ and $\Gamma_2=-(\nu-\mu)$.

V. ARBITRARY INITIAL STATE OF ATOM B

If the initial state of atom B is arbitrary, say $|\phi\rangle=\cos\theta|g_L\rangle_B+e^{i\vartheta}\sin\theta|g_R\rangle_B$ with θ and ϑ being arbitrary. Eq. (23) in the main text becomes:

$$\begin{aligned}
\widetilde{c}_{e,\omega}(t) &= -i(\alpha\cos\theta+\beta e^{i\vartheta}\sin\theta)\frac{g_2}{\eta}e^{\lambda t}\sinh\eta t,\\
\widetilde{c}_{1,\omega}(t) &= (\alpha\cos\theta+\beta e^{i\vartheta}\sin\theta)(\frac{2\rho-\lambda}{2\eta}e^{\lambda t}\sinh\eta t+\frac{1}{2}e^{\lambda t}\cosh\eta t-\frac{1}{2}e^{2\rho t})+\alpha\cos\theta e^{2\rho t},\\
\widetilde{c}_{2,\omega}(t) &= \beta\cos\theta e^{2\rho t},\\
\widetilde{c}_{3,\omega}(t) &= \alpha e^{i\vartheta}\sin\theta e^{2\rho t},\\
\widetilde{c}_{4,\omega}(t) &= (\alpha\cos\theta+\beta e^{i\vartheta}\sin\theta)(\frac{2\rho-\lambda}{2\eta}e^{\lambda t}\sinh\eta t+\frac{1}{2}e^{\lambda t}\cosh\eta t-\frac{1}{2}e^{2\rho t})+\beta e^{i\vartheta}\sin\theta e^{2\rho t}.
\end{aligned} \quad \text{(S17)}$$

Eq. (24) and (25) in the main text remain unchanged. For simplicity, we only consider the case of $\Delta = 0$, $\delta_\omega = 0$ and infinite time limit. Then according to Eq. (25) in the main text the tripartite state is,

$$|\Psi\rangle_{ABP} = \frac{1}{\sqrt{2}}(|g_L\rangle_A|g_R\rangle_B - |g_R\rangle_A|g_L\rangle_B) \otimes (\cos\theta|R\rangle - \mathrm{e}^{\mathrm{i}\vartheta}\sin\theta|L\rangle). \quad \text{(S18)}$$

In our non-monochromatic incident light case, if the initial state of atom B is $|\phi\rangle = |g_R\rangle$, then Eq. (28) in the main text becomes:

$$|\psi(t)\rangle = cc_1(t)|g_R\rangle_A|g_R\rangle_B|R\rangle + cc_2(t)|g_L\rangle_A|g_R\rangle_B|L\rangle + cc_3(t)|g_R\rangle_A|g_L\rangle_B|L\rangle. \quad \text{(S19)}$$

According to this, if the initial state is the arbitrary case $|\phi\rangle = \cos\theta|g_L\rangle_B + \mathrm{e}^{\mathrm{i}\vartheta}\sin\theta|g_R\rangle_B$, then Eq. (28) in the main text becomes:

$$\begin{aligned}|\psi(t)\rangle &= |g_L\rangle_A|g_R\rangle_B(cc_3(t)\cos\theta|R\rangle + cc_2(t)\mathrm{e}^{\mathrm{i}\vartheta}\sin\theta|L\rangle) + |g_R\rangle_A|g_L\rangle_B(cc_2(t)\cos\theta|R\rangle + cc_3(t)\mathrm{e}^{\mathrm{i}\vartheta}\sin\theta|L\rangle)\\ &+ cc_1(t)(\cos\theta|g_L\rangle_A|g_L\rangle_B|L\rangle + \mathrm{e}^{\mathrm{i}\vartheta}\sin\theta|g_R\rangle_A|g_R\rangle_B|R\rangle). \quad \text{(S20)}\end{aligned}$$

After a sufficiently long time, say $t > t_{start}$, the assumption $cc_1(t) \approx 0$ and $cc_2(t) \approx -cc_3(t)$ are appropriate. The normalized tripartite state is:

$$|\Psi\rangle_{ABP} \approx \frac{1}{\sqrt{2}}(|g_L\rangle_A|g_R\rangle_B - |g_R\rangle_A|g_L\rangle_B) \otimes (\cos\theta|R\rangle - \mathrm{e}^{\mathrm{i}\vartheta}\sin\theta|L\rangle). \quad \text{(S21)}$$
